# 

**Published:** 1881-12

**Authors:** 


### Pamphlets.

The galvanic accumulator for storing dynamical electricity for cautery and illuminating purposes. By Louis Elsberg, A.M., M.D. Reprint from the proceedings of the New York Academy of Medicine.

1. Remarkable change in the color of the hair, from light blonde to black in a patient, while under treatment by pilocarpin. Report of a case of pyelonephritis, with unusually long anuria.

2. Case of membranous croup treated successfully by pilocarpin. By D. W. Prentiss. A.M., M.D.

Uterine massage as a means of treating certain forms of enlargement of the womb. By A. Reeves Jackson, A.M., M.D.

